# Diagnostic Accuracy of Digital and Conventional Radiography in the Detection of Non-Cavitated Approximal Dental Caries

**DOI:** 10.5812/iranjradiol.6747

**Published:** 2012-03-25

**Authors:** Farida Abesi, Alireza Mirshekar, Ehsan Moudi, Maryam Seyedmajidi, Sina Haghanifar, Nima Haghighat, Ali Bijani

**Affiliations:** 1 Dental Materials Research Center, Department of Oral and Maxillofacial Radiology, Dental Faculty, Babol University of Medical Sciences, Mazandaran, Iran; 2 Student Research Committee of Babol University, Dental Faculty, Babol University of Medical Sciences, Mazandaran, Iran; 3 Non-Communicable Pediatric Disease Research Center, Babol University of Medical Sciences, Mazandaran, Iran

**Keywords:** Radiography, Dental, Digital, Dental Caries, Diagnosis

## Abstract

**Background:**

Radiography plays an important role in the detection of interproximal caries.

**Objectives:**

The aim of the present study was to determine diagnostic accuracy of chargecoupled devices (CCD), Photo Stimulable Phosphor (PSP) and film radiography in detecting non-cavitated caries.

**Patients and Methods:**

Seventy-two non-cavitated approximal surfaces of extracted human posterior teeth were radiographed under standardized conditions using three intraoral modalities: CCD Dixi3 (Planmeca, Finland), PSP Digora PCT (Soredex, Finland),and E-speed film (Kodak, USA). Radiographs were interpreted by four observers and caries lesions were classified as sound (R0), restricted to enamel (R1), reaching the dentinoenamel junction (DEJ) and the outer half of the dentin (R2) and the inner half of the dentin (R3). The teeth were subsequently sectioned for histological analysis which served as the gold standard for radiographic examination.

**Results:**

Microscopic examinations showed that the distribution of caries were 63.9% sound, 18.1% enamel, 9.7% DEJ and outer half of the dentin and 8.3% into the inner half of the dentin.

The sensitivity and specificity of film, CCD and PSP for the detection of enamel caries were 38% and 98%; 15% and 96 %; and 23% and 98%, respectively.

The sensitivity and specificity of film, CCD and PSP for the detection of both dentin and enamel caries were 55% and 100%; 45% and 100% ; and 55% and 100%, respectively.

**Conclusions:**

The results demonstrated that the diagnostic accuracy of digital images is similar to that of conventional film radiography in the detection of non-cavitated approximal caries.

## 1. Background

Radiography along with clinical findings is considered as a routine diagnostic approach for caries detection. Unfortunately, there is not a quite sensitive and precise method available for the early detection of caries at the present time. Accurate diagnosis of primarily, non-cavitated caries is a matter of high significance since disease progression can be easily halted at this stage, and tooth structure can also be preserved with minimal invasion only by utilizing conservative and not by restorative treatment [1][2][3][4]. Although researchers are seeking tools with sufficient sensitivity and specificity for this purpose, different findings have demonstrated that none of these new methods and common available devices is able to detect caries in all dental surfaces [5][6]. Nevertheless, radiography still remains as the most common approach [7][8].

Over the past recent years, diagnostic accuracy of digital radiography systems for caries detection has been compared mutually and with conventional film system [9][10][11][12][13].

Some studies consider the image quality of radiographic films comparable to that of the systems with charge-coupled devices (CCD) [10] and to the ones that use storage phosphor plates [14][15]. Other studies reported superiority of the systems with storage phosphor plates over conventional radiographs and over systems with CCD [16][17][18][19].

There are also researches demonstrating better diagnostic accuracy of conventional film radiographs compared to digital systems [20]. However, few studies have been implemented on non-cavitated interproximal caries detection [21][22][23].

Furthermore, sensitivity of imaging systems is assumed to be more for cavitated caries diagnosis.

## 2. Objectives

In line with the above bodies of evidence, the present study was conducted to determine diagnostic accuracy of CCD Dixi3 and PSP plates versus film radiography in detecting non-cavitated approximal caries.

## 3. Patients and Methods

This diagnostic accuracy study was carried out on 48 extracted human posterior teeth (72 dental surfaces) with both sound and carious approximal surfaces in the Dental Material Research Center of Babol University. In visual inspection, carious surfaces had varying degrees of demineralization appearing as chalky white or brownish discoloration areas. Exclusion criteria included those with restoration on approximal surfaces, extensive buccal or lingual caries, dental wear, presence of fractures or anomalies and cavity in approximal surfaces.

In the present study, the teeth were embedded in blocks of Paris plaster in an anatomical position to establish approximal surfaces in contact.

**Figure 1:**
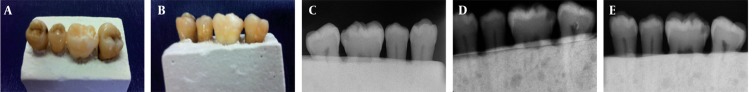
A and B, Depicting the clinical view of the dental block; C, E-speed film radiograph; D, CCD radiograph; E, PSP radiograph

Each block consisted of four teeth, in which two interdental contacts including six interdental surfaces existed; two non-contacted surfaces were not considered in the study (Figure 1A-E).

Conventional and digital images of the teeth were acquired using three intraoral modalities: E-speed film (Eastman Kodak, Rochester, NY), CCD (Dixi3, Planmeca, Finland): 19-38 microns pixel size and 13-26 line-pairs/ mm (lp/mm) resolution and PSP receptor (Digora PCT, Soredex, Finland): 85-167 micron pixel size and 6-8 lp/mm resolution. Standardized conditions were used: DC intraoral x-ray unit, (Minray, Soredex, Finland) at 60 kVp, 8 mA and 0.2 s for E-speed, 10 mm tooth-receptor distance, 30 cm target-to-receptor distance, rectangular collimation, paralleling technique. A 24-mm plexy-glass plate was placed between the tube extension and the teeth to simulate soft tissue. A stabilizer device was used to maintain the projection geometry. Then all the films were simultaneously developed by an automatic processor (Hope, Dental Max, USA) using Tetenal developer and fixer solution, according to the manufacturer’s instructions. For digital exposure, it should be noted that all adjustment conditions for teeth and radiography apparatus were similar to before, the only difference was in the exposure time which was reduced to approximately 0.08 second.

### 3. 1. Radiographic Evaluation

Each of the digital and conventional radiographic images was allocated a code. Four oral and maxillofacial radiologists acted as observers, all of whom had at least five years experience in caries diagnosis on digital images and film. The observers were allowed to use the image enhancement facilities as they wished. First, pictures provided by conventional radiography were observed in a quiet dark space on the view box. Then digital images were examined one by one in a predetermined random order on a DFX 17-inch CRT monitor (Samsung and Sync-Master 1793) with high resolution and no time limitation, in a semi-dark room.

**Figure 2:**
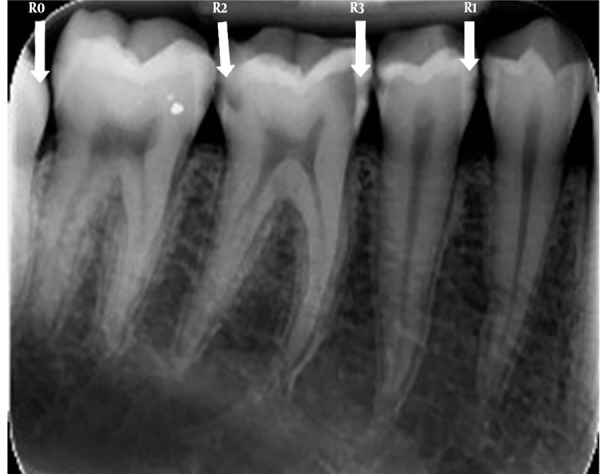
Classification of caries according to the depth of caries in radiography. Sound (R0), restricted to the enamel (R1), reaching the dentinoenamel junction, reaching the outer half of the dentin (R2) and the inner half of the dentin (R3)

Radiographs were encoded according to the following (Figure 2):

R_0_, Sound; R_1_, Radiolucency restricted to the enamel; R_2_, Radiolucency reaching the dentino-enamel junction (DEJ) and the outer half of the dentin; R_3_, Radiolucency into the inner half of the dentin.

In each viewing session, observers assessed images of one type of modality simultaneously so that they announced one opinion consensually. A period of two weeks separated each viewing session.

### 3.2. Histological Examination

**Figure 3:**
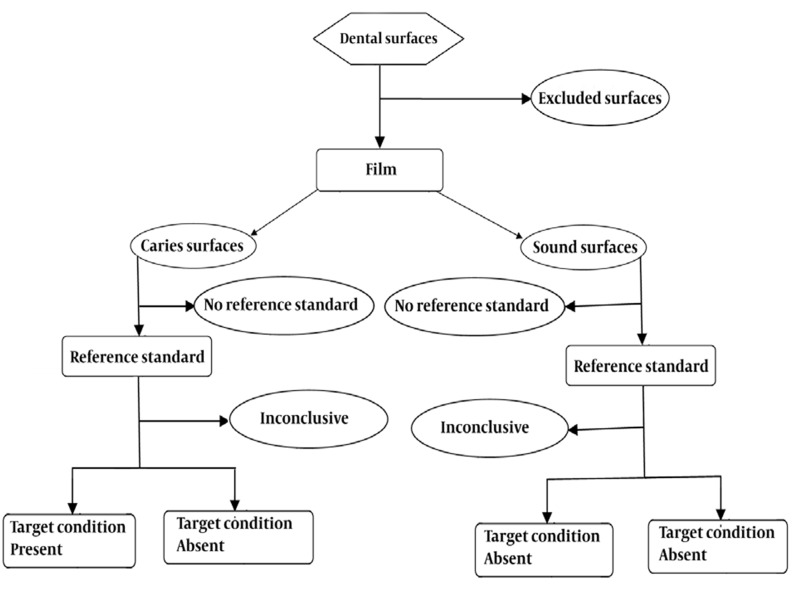
Flow diagram for conventional radiography (film) in enamel caries

Variables were validated using histological section as gold standard. In this regard, teeth were individually embedded in a triplex transparent acrylic (Ivoclar Viva-dent, Liechtenstein) and serially sectioned, using hard-tissue sectioning apparatus, Acuatum 50 (43 TCA, Ball Erud, Denmark) with a 400 microns diamond blade and 300-3000 rpm spindle speed, into 400 μm thick sections in the mesio-distal direction so as to pass through the height of contour (Figure 3).

Dental sections were simultaneously examined in both sides by an observer (oral and maxillofacial pathologist) under a stereomicroscope (Olympus SZX, Optical Co, Ltd) with 10 × magnification. A carries lesion was confirmed when demineralization was observed as an opaque white or dark-brown discoloration under the height of contour.

Data were exported to SPSS 17 statistical software. Two analyses were done. The first analysis was performed to detect all lesions in approximal surfaces: sound surfaces (absence of caries = R_0_) versus surfaces with lesions (presence of caries = R_1_, R_2_, R_3_) so that the sensitivity, specificity, accuracy, positive predictive value and negative predictive value was carried out for these binary data (determination of presence or absence of caries).

And the second analysis was Kappa coefficient that was performed for each modality according to the depth of caries. The significance level was set at P < 0.05.

## 4. Results

Histological examination of the 72 dental surfaces showed that 63.9% of the surfaces were noncarious, 18.1% of the caries confined to the enamel, 9.7% extended into the DEJ and the outer half of the dentin and 8.3% into the inner half of the dentin.

In addition, teeth with caries extended into the inner half of the dentin were excluded from the study.

As presented in Table 1, the highest and lowest sensitivity for enamel carries was related to film and CCD, respectively. Furthermore, kappa coefficient between film and gold standard was 0.61 (P = 0.0001), 0.47 for PSP (P = 0.0001) and 0.31 for CCD (P = 0.0001).

**Table 1:** Indices of Diagnostic Test Accuracy for Each Radiographic Modality for Enamel Caries

	**Sensitivity, %, (95% CI a)**	**Specificity, %, (95% CI)**	**PPV a, %, ** **(95% CI)**	**NPV a, %, ** **(95% CI)**	**LR+ a, ** **(95% CI)**	**LR- a, ** **(95% CI)**
Film	38 (12-65)	98 (94-100)	83 (54-100)	87 (78-95)	20.38 (2.60-159)	0.63 (0.41-0.97)
CCD a	15 (0-35)	96 (91-100)	50 (1-99)	82 (73-92)	4.08 (0.63-26.29)	0.88 (0.69-1.12)
PSP a	23 (0-46)	98 (94-100)	75 (33-100)	84 (75-93)	12.23 (1.38-108)	0.78 (0.58-1.06)

^a^ Abbreviations: CCD, charge-coupled devices; CI: confidence interval; LR+, positive likelihood ratio; LR-, negative likelihood ratio; NPV, negative predictive value; PPV, positive predictive value; PSP, photo stimulable phosphor

In the end, indicators of diagnostic test accuracy were estimated for each modality for both dentin and enamel caries which is mentioned in Table 2. So as shown, no significant difference was detected between conventional and digital radiography in terms of sensitivity and specificity for the mentioned caries detection.

**Table 2:** Indices of Diagnostic Test Accuracy for Each Radiographic Modality for Both Dentin and Enamel Caries

	**Sensitivity, %, (95% CI a)**	**Specificity, %, (95% CI)**	**PPV **a**, %, (95% CI)**	**NPV **a**, %, (95% CI)**	**LR+ **a**, (95% CI)**	**LR_ **a**, (95% CI)**
Film	55 (33-77)	100 (92-100)	100 (72-100)	84 (74-93)	-	0.45 (0.28-0.73)
CCD **a**	45 (23-67)	100 (92-100)	100 (66-100)	81 (70-91)	-	0.55 (0.37-0.82)
PSP **a**	55 (33-77)	100 (92-100)	100 (72-100)	84 (74-93)	-	0.45(0.28-0.73)

^a^ Abbreviations are explained in the footnote of Table 1

## 5. Discussion

We conducted this in vitro study, which permitted the histologic examination of the approximal surfaces as the ultimate validation criterion. The range of sensitivity values for diagnosing enamel lesions was 15-38%, meaning that all systems performed poorly in detecting small enamel lesions. This finding is in accordance with the previous studies done by Wenzel and Haiter-Neto, demonstrating system failure in small lesion detection ([24]-[26]). Of course cone beam CT (CBCT) scan was also used in addition to digital modalities in the latter study.

Our study findings demonstrated no significant differ-ence between digital and conventional radiographic modalities in the detection of non-cavitated interproximal caries.

In line with this, Aberu study on diagnostic accuracy of CCD (RVG UI) digital radiography and Ekta Speed plus film radiographs displayed no significant difference between the two imaging systems in determining interdental caries [21]; whereas, in Uprichard’s study on the diagnostic accuracy of digital systems and conventional radiography in detecting approximal caries during mixed dentition, the diagnostic accuracy of radiographic film was reported to be more remarkable than digital CCD [22]. In the explanation of this contradiction, it can be noted that Uprichard’s study consisted of both cavitated and noncavitated carious teeth, while in the present study, only non-cavitated teeth were used.

Among the investigations performed on non-cavitated caries lesions, in two studies by Pontual and Haiter-Neto, the performance of PSP plate was reported to be similar to that of the film for detection of approximal enamel caries [2][22] which is in consistence with the present research. Whereas, in Wenzel’s study, Diagora Optime super-resolution displayed higher sensitivity than almost all the other modalities but had a lower specificity than all other modalities, among which there was no differences [24]. This contradiction may be due to different types of detectors used in the mentioned study and the present research; as E-Speed film as well as Digora PCT were used in our study with 30 s scanning time and 6 lp/mm spatial resolution, but about half the radiation dose compared to film; while in Digora Optime, which was applied in Wenzel’s study, spatial resolution is 15 l p/mm and scanning time 8s with the dose approximately equal to film, meaning that there is a significant dose increase [24].

Moreover, CCD Dixi3 was applied in the present study with a spatial resolution rate more than Dixi2 (16 lp/mm);however, unlike the expectation, there is a poor agreement between CCD findings and the gold standard, so as in CCD images, there were more noise levels in comparison to the two other modalities (despite the use of the anti-noise button (item) in the software).

Some studies have demonstrated that despite advances in spatial resolution in new generations of solid states, the difference between modalities was still non-significant for the overall accuracy [26][27][28].

The strong point of our study was that there was a consensus among four oral and maxillofacial radiologists as observers in each observation however the limitation of this study was the lack of repeated observations by each observer. It seems that other factors such as proper range of radiation dose, scatter radiation levels and even contrast level can influence the image quality in digital images.

In conclusion, our study demonstrated that in noncavitated lesions the diagnostic accuracy improved more with the depth of the lesions. In enamel lesions, film (conventional system) had better results, although there was no significant difference between conventional and digital systems.
